# Wnt16: a new potential therapeutic target for osteoporotic non-vertebral fracture treatment

**DOI:** 10.3389/fragi.2026.1768566

**Published:** 2026-03-09

**Authors:** Chen Tianpeng, Xia Qishui, Yang Fo, Wu Mingjun, Hu Haibo, Jin Jiuchu, Shi Xiaolin, Jiang Gongtao

**Affiliations:** 1 Department of Orthopedics, Nanchang Hongdu Hospital of Traditional Chinese Medicine Affiliated to Jiangxi University of Chinese Medicine, Nanchang, Jiangxi, China; 2 Department of Orthopedics, The Second Affiliated Hospital of Zhejiang Chinese Medical University, Hangzhou, Zhejiang, China

**Keywords:** BMD, bone, non-vertebral fracture, osteoporosis, Wnt16

## Abstract

Osteoporotic non-vertebral fractures are a major clinical burden, with cortical bone impairment being a key pathogenic factor often overlooked in traditional treatments. This review aims to synthesize current evidence on the role of Wnt16 (a non-canonical Wnt ligand) in regulating cortical bone and its potential as a therapeutic target for osteoporotic non-vertebral fractures. We systematically review literature on Wnt16 in senile, postmenopausal, and glucocorticoid-induced osteoporosis, focusing on its mechanisms of action: (1) regulating bone mineral density via genetic associations with GWAS-identified loci; (2) reducing cortical bone porosity and increasing thickness; (3) dual regulation of osteoblasts (via JNK/β-catenin pathways) and osteoclasts (via OPG-dependent/independent NF-κB pathways). Wnt16 has been shown to improve bone density and reduce non-vertebral fracture risk in preclinical models, though conflicting findings exist regarding its full compensation for glucocorticoid-induced bone loss. We conclude that Wnt16 is a promising target for non-vertebral fracture prevention, with Notum inhibitors emerging as potential therapeutic agents. This review provides a comprehensive framework for future clinical and translational research.

## Introduction

The risk of fracture is greatly increasing for patients with osteoporosis due to the decrease of bone mass, destruction of bone microstructure and increase of bone fragility (Cauley, 2017). People over the age of 50 have twice the risk of fracture as people under the age of 50 in 10 years (Heuchemer et al., 2020). The wrist, hip and vertebra are the most common sites for osteoporotic fragility fractures. In general, osteoporotic fractures are mainly divided into vertebral fractures and non-vertebral fractures. The non-vertebral fractures mainly refer to hip, distal radius, rib and pelvis fractures (Curtis et al., 2017). Fragility fractures, particularly those non-vertebral fractures are associated with increased mortality and significant morbidity (Zeytinoglu et al., 2017; Hochberg, 2008; Liu et al., 2019). At present, anti-osteoporosis drugs mainly include calcium, vitamin D, bisphosphate, teriparatide, estrogen and receptor activator of nuclear factor-κb ligand (RANKL) inhibitors. However, the therapeutic effects of the current clinical used anti-osteoporosis drugs are far from satisfactory, particularly in non-vertebral fractures (Khalid et al., 2018). Therefore, a new drug is urgently needed to prevent osteoporotic non-vertebral fractures.

Vertebral fractures aremainly caused by the decrease of cancellous bone content other than the thinning or brittleness of cortical bone (Jin et al., 2019), the signal pathways involved in the regulation of cortical and cancellous bone may be different. Studies on Pyle’s disease confirmed this speculation. In patients with Pyle’s disease, the trabeculae of metaphysical bone is uniform, but the cortical bone of bone stem is obviously reduced (Kiper et al., 2016). The signaling pathways involved in osteoporotic fractures mainly include the classical Wnt signaling pathway and non classical Wnt signaling pathway. The non classical Wnt signaling pathway includes Wnt16. Because Wnt16 mainly regulates the bone cortex, which has more potential to prevent osteoporosis and non vertebral fractures. This study mainly reviews the mechanism and role of Wnt16 in osteoporosis. Wnt signaling pathway, which includes the classical and non-classical ones, plays an important role in bone homeostasis regulation (Kobayashi et al., 2016). In the classical Wnt signaling pathway, the Wnt ligand induces the expression of transcription factor osterix by activating the β-center signal, thus promoting bone formation. At the same time, this signal increased the formation of osteoprotegerin (OPG) and inhibited bone resorption (Maeda et al., 2013).

**FIGURE 1:**
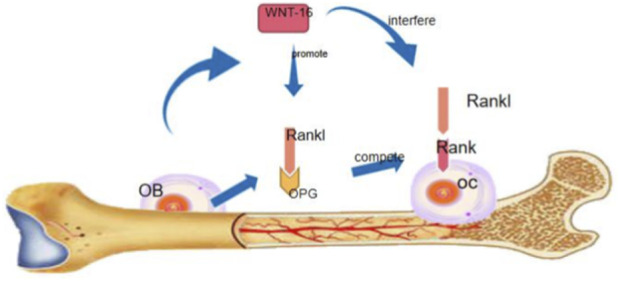
Wnt16 can inhibit the expression of rank receptor and interfere with the binding of rank receptor with rankle, and inhibit the differentiation and maturation of osteoclasts. Wnt16 can also indirectly inhibit osteoclast differentiation through the expression of OPG.

In the nonclassical Wnt signaling pathway, Wnt5a inhibits the trans-activation of PPAR-γ of co-repressor complex about kinase 1-Nemo-like kinase signal transduction activated by calcium-calmodulin-dependent protein kinase II-TGF-β, and induces the expression of Runx2, thus promoting osteoblast formation (Okamoto et al., 2014). In a recent study, another non-classical Wnt signal pathway, Wnt16, which is independent of β-center signal, was discovered. Wnt16 is a member of the family, mainly expressed in cortical bone and periosteum (Gori et al., 2015). It is found that Wnt16 secreted by osteoblasts can directly act on the receptor activator of nuclear factor-b (RANK) receptor of osteoclasts, interfere with the combination of RANKL and RANK receptor, and inhibit the differentiation of osteoclasts. On the other hand, Wnt16 competitively inhibits the binding of RANKL to RANK receptor by promoting the production of osteoprotegerin in osteoblasts, thus inhibiting bone resorption on the cortical bone (Figure 1) (Baron and Kneissel, 2013). In terms of the effect of Wnt16 on mesenchymal stem cells, Wnt promotes the differentiation of human perivascular stem/stromal cells (HPSCs) osteoblasts by activating JNK, and the use of specific JNK inhibitors can inhibit the up-regulation of osteogenesis induced by Wnt16, which verifies that the osteogenic effect of Wnt16 in HPSCs in vito depends on JNK signal pathway (Shen et al., 2018). Wnt16 has dual regulatory effects. It can not only promote osteogenesis but also inhibit osteoclast. Wnt5a can promote both osteogenesis and osteoclast, and the outcome depends on the bone microenvironment. Compared with the classical Wnt signal, Wnt16 has more advantages in regulating cortical bone because Wnt16 mainly expresses in cortical bone.

## The mechanism of Wnt16 in preventing osteoporosis and non-vertebral fracture

### Wnt16 regulates bone mineral density

Bone mineral density is closely related to osteoporotic fracture. Bone mineral density-related genes play a decisive role in fracture. Their research confirmed the 2p16.2 (SPTBN1), 7q21.3 (SHFM1), 10q21.1 (MBL2/DKK1), 11q13.2 (LRP5), and 18p11.21 (FAM210A) loci, and observed an increased signal at SOST, CPED1/Wnt16, FUPB3, DCDC5, RPS6KA5, STARD3NL, and CTNNB1. Lastly, we added the 6q22.33 (RSPO3), 6q25.1 (ESR1), 7p12.1 (GRB10/COBL), and 21q22.2 (ETS2) loci into the list of novel fracture loci.

The data of genome-wide association study (GWASs) has found 90 loci, such as SOST, Wnt16, ESR1 and RANKL were strongly correlated with bone mineral density, which regulates the thickness of bone cortex (Lovšin et al., 2018; Trajanoska and Rivadeneira, 2019; Trajanoska et al., 2018). Wnt16 is of great significance in preventing osteoporosis and non-vertebral fracture. Karol Estrada’s research found that bone mineral density (BMD) changes are highly polygenic and complex (Estrada et al., 2012). Wnt16rs3801387, a BMD locus, is associated with the risk of low-energy fracture, which clarifies the pathophysiological mechanism of fracture susceptibility. See Table 1.

**TABLE 1:** Genetic loci and associated genes regulating bone metabolism and cortical bone properties.

Locus	Genes involved	Association with bone metabolism
2p16.2	SPTBN1	Regulates bone mineral density (BMD)
7q21.3	SHFM1	Correlates with cortical bone thickness
10q21.1	MBL2/DKK1	Inhibits canonical Wnt signaling, reduces BMD
11q13.2	LRP5	Enhances Wnt signaling, increases cortical density
18p11.21	FAM210A	Modulates BMD and fracture susceptibility
1p36.12	CPED1/Wnt16	Key regulator of cortical bone thickness/porosity
6q22.33	RSPO3	Promotes Wnt signaling, improves bone mass
6q25.1	ESR1	Estrogen-mediated osteoprotection, regulates Wnt16

### Wnt16 affects the porosity of bone cortex

Cortical bone impairment accounts for 80% of non-vertebral fractures, with thickness and porosity being critical determinants of bone strength. Preclinical studies have consistently shown that Wnt16-deficient mice exhibit significantly thinner cortical bone and increased porosity, leading to a marked elevation in fracture risk (Koller et al., 2013; Okamoto et al., 2014; Movérare-Skrtic et al., 2014). Two key mechanisms underlie this phenomenon: first, increased cortical porosity reduces bone’s ability to withstand mechanical stress; second, thinning of the cortical bone directly impairs bone strength. Clinical studies in elderly women with hip fractures (a common non-vertebral fracture) have confirmed that cortical bone thinning and porosity increase independently of BMD (Epstein, 2007; Sundh et al., 2017), highlighting the clinical relevance of Wnt16-mediated cortical bone regulation. Unlike trabecular bone loss, which is often the focus of traditional osteoporosis therapies, cortical bone loss is the primary driver of non-vertebral fracture risk in the elderly (Zebaze et al., 2010). Thus, strategies targeting Wnt16 to increase cortical bone thickness and reduce porosity may offer a novel approach to preventing non-vertebral fractures, beyond mere BMD enhancement.

## Mechanism of Wnt16 in preventing non vertebral fracture of osteoporosis

### Wnt16 and bone cortex

The fragility of cortical bone is the main factor causing osteoporotic non-vertebral fracture. However, little is known about the regulatory mechanism of cortical thickness and density (Goltzman, 2019). Claes Ohlsson et al. studied the model of Wnt16 inactivated mice. The results showed that the cortical thickness of Wnt16 inactivated mice decreased significantly compared with control mice (P < 0.05), which was caused by the increase of bone resorption and the decrease of periosteum. This suggests that Wnt16 is a key regulator of cortical bone growth and development and a major determinant of cortical bone thickness and non-vertebral fractures (Ohlsson et al., 2018a). Therefore, Wnt16 may be a key target for the prevention of osteoporosis and non-vertebral fractures. Because Wnt16 is a potential target for the prevention of osteoporosis and non-vertebral fractures (Grigorie and Leerner, 2018), Sophia Mov Skrtic further demonstrated the role of Wnt16 in intraosseous homeostasis by inducing high Wnt16 expression in mice induced by tamoxifen. They found that in Tamoxifen induced Wnt16 overexpression mice, periosteal formation increased and the cortical bone significantly thickened, but the trabecular number did not increase. This suggests that Tamoxifen Wnt16 may occur primarily in the cortical bone. Sophia also found a significant increase in Wnt16 mRNA expression in the cortical bone of the FEMUR. This finding further confirms that Wnt16 modulates bone mass in the bone cortex. In another study, the phenomenon was also explained: researchers deleted the Wnt16 gene altogether. The cortex of the mice was very thin, but the long bones and trabeculae of the spine were normal. Wnt16 acts on osteoclast precursors in cortical bone and negatively regulates osteoclast formation, resulting in an increase in cortical rather than trabecular bone. Therefore, we hypothesize that Wnt16 acts on cortical bone and plays an active role in preventing cortical bone loss. Wnt16 may be a potential target for the prevention of osteoporosis, non-vertebral fractures,and providing research direction for drug development.

### Wnt16 and osteogenesis

Wnt16 exerts context-dependent effects on osteogenesis, with conflicting findings attributed to differences in experimental models and signaling pathways. In human perivascular stem/stromal cells (HPSCs), Wnt16 promotes osteoblast differentiation via activation of the JNK signaling pathway (Shen et al., 2018); this effect is abrogated by specific JNK inhibitors, confirming the pathway’s necessity. In contrast, in MC3T3-E1 preosteoblasts, Wnt16 inhibits mineralization by suppressing the canonical β-catenin pathway (Jiang et al., 2014), an effect reversed by DKK1 (a canonical Wnt inhibitor). These findings suggest that Wnt16 acts as a context-dependent agonist/antagonist in the canonical Wnt pathway: it inhibits β-catenin-mediated mineralization in MC3T3-E1 cells but promotes osteogenesis via non-canonical JNK signaling in HPSCs. Additionally, Wnt16 can enhance osteoblast differentiation by competing with canonical Wnt ligands (e.g., Wnt3a), further supporting its pathway-specific regulatory role in bone formation.

### Wnt16 and osteoclast

Osteoclast formation was promoted by co-culture of Wnt16(Wnt16^−/−^) with osteoclasts at the site of 1, 25-dihydroxyvitamin D3[1α, 25(OH)2D3]. This study showed that Wnt16 secreted by osteoblasts has a negative regulatory effect on osteogenesis. Wnt16 activates MC3T3-E1 cells. Wnt (mouse preosteoblast)/β-catenin signal induces OPG expression, then inhibits RANK-RANKL interaction and directly inhibits osteoclast differentiation. However, in OPG knockout mice cocultured with 1α, 25-dihydroxyvitamin D3[1α, 25(OH)2D3], Wnt16 can still inhibit osteoclast formation induced by RANKL and inhibit osteoclast formation induced by NF-κb signal transduction pathway (via the combination of NF-κb, RANK and RANKL stimulating NF-κb inhibitor kinase β (IKK β)) phosphorylation in osteoclast precursors, nuclear factor kappa B induced nf-κb to enter nf-κb into the nucleus (Wang and Guo, 2015) NFATC1. Further, Wnt16 is an independent OPG, which directly affects the anti-inflammatory effect of RANKL induced by the precursor of osteoclasts. Therefore, Wnt16 indirectly increases the expression of OPG in osteoblasts and directly acts on osteoclast precursors, inhibition of osteoclast formation and Wnt16 stimulation of ion activation in osteoclast precursor cells is a non-classical Wnt signaling pathway, while osteoblasts are stimulated by both active classical signaling pathways and non-classical Wnt signaling pathways.

## Prevention and treatment of various osteoporosis non-vertebral fractures with SanWnt16

### Wnt16 in osteoporosis and non-vertebral fracture caused by GC

Glucocorticoid (GC) use significantly increases the risk of osteoporosis and non-vertebral fractures, primarily by inhibiting the canonical β-catenin pathway and upregulating Wnt inhibitors (e.g., SOST) (Trajanoska and Rivadeneira, 2019). GC also directly promotes osteoclast proliferation and osteoblast apoptosis, leading to cortical bone loss (Whittier and Saag, 2016). Studies on Wnt16’s role in GC-induced osteoporosis have yielded conflicting results: Hildebrand’s study showed that recombinant Wnt16 restores osteoblast function in GC-treated cells (Hildebrandt et al., 2018), and Wnt16 transgenic mice exhibit higher BMD than wild-type mice after GC exposure (Ohlsson et al., 2018b); however, another study found that Wnt16 overexpression fails to prevent cortical bone loss in GC-treated mice (Alam et al., 2018). These discrepancies likely stem from three factors: (1) GC dosage and duration (short-term low-dose GC may be counteracted by Wnt16, while long-term high-dose GC causes irreversible bone damage); (2) model type (global Wnt16 overexpression vs. conditional overexpression in osteoblasts); (3) outcome measures (BMD assessment alone vs. combined cortical porosity/thickness analysis). We hypothesize that Wnt16 can partially mitigate GC-induced bone loss by preserving cortical bone thickness and inhibiting bone resorption, but cannot fully compensate for the direct cytotoxic effects of GC on osteoblasts and osteoclasts. Further studies with standardized GC regimens and tissue-specific Wnt16 modulation are needed to resolve this controversy.

### Wnt16 prevents and treats postmenopausal osteoporosis non-vertebral fracture

Postmenopausal older women are at high risk for osteoporosis, and fractures can be devastating for them (Black and Rosen, 2016). The study by Tomasz Mitek et al. found significant differences in Wnt16 RS2908004 gene polymorphism among postmenopausal women with osteoporosis, especially those at high fracture risk (Mitek et al., 2019). Estrogen can increase bone mineral density and reduce fracture risk in postmenopausal osteoporosis patients by increasing trabeculae and inhibiting cortical bone resorption (Levin et al., 2018). To investigate the association between estrogen and Wnt16, Henry Todd found that Wnt16 increased after intervention with Estradiol and Tamoxifen, suggesting that the risk of estrogen preventing fractures is mediated by Wnt16. In Henry Todd’s study, bone cortices were more than 10 times as strong as those in ESR1 deficient wild-type mice treated with Tamoxifen and ESR1, this suggested that Wnt16 can compensate for bone loss due to ESR1 deficiency. However, estrogen and Wnt16 have independent osteoprotective effects in the study of Mov rare Skrtic. After ovariectomy, the bone mineral density of Wnt16-overexpressed transgenic mice decreased to the same level as that of wild-type ovariectomized mice (Chamkasem and Toniti, 2015). In addition, estrogen still enhanced bone strength in Wnt16-deficient transgenic mice. Although it is debatable whether the protective effect of estrogen on bone is related to Wnt16, Wnt16 can increase the cortical bone and prevent fracture risk in ovariectomized mice (Almeida et al., 2017). In conclusion, Wnt16 may be a potential target for the prevention of Postmenopausal osteoporotic fractures.

### Wnt16 and senile osteoporosis non-vertebral fracture

Age and gender are important influencing factors of osteoporosis and fracture (Sims et al., 2002). Wnt16 plays an important role in bone homeostasis in patients with osteoporosis and postmenopausal osteoporosis but lacks the effect of age and gender on Wnt16. Xiangshen Long studied the gender and age differences of Wnt gene expression in human bone marrow stromal cells. They only found that there was a significant difference between Wnt16 and gender, and the expression in men was higher than that in women. There was no significant difference in the expression level of Wnt16 in rats of different ages (Wang, 2010; Shen et al., 2009). In Henry Todd’s study, it was found that the expression of Wnt16 in bone cortex decreased with age, but it did not change with age in the kidney. Therefore, we speculate that the difference between the above two experimental results may be due to the detected tissue differences. Wnt16 is mainly expressed in cortical bone and periosteum, so we tend to the latter conclusion. Wnt16 expression decreases with age. This reveals another internal reason for the high prevalence of osteoporosis and high risk of fracture in the elderly. Wnt16 may provide guidance for the study of senile osteoporosis. On the other hand, Wnt16 may play a role in bone growth and development, which is worthy of further study. The decrease of activity and gastrointestinal absorption with age may be the main cause of senile osteoporosis. Estrogen deficiency is an important cause of osteoporosis in elderly women. In Henry Todd’s study, the relationship between Wnt16 and the above factors has been verified. They found that the expression of Wnt16 varied with age and gender. The expression of Wnt16 in cortical bone of male rats decreased with age, but it was up-regulated in bone marrow. In female rats, Wnt16 decreased in cortical bone and bone marrow. This difference may be due to differences in estrogen levels. In addition, they found that the reduction of mechanical load did not affect the expression level of Wnt16, but estrogen deficiency resulted in insufficient expression of Wnt16. On the contrary, estrogen Wnt16 expression was up-regulated. This suggests that Wnt16 is involved in some bone mass regulation (Movérare-Skrtic et al., 2019).

## Discussion

### Clinical relevance: Wnt16 as a fracture risk predictor

Bone mineral density (BMD) has long been used to predict fracture risk, but it is less sensitive for non-vertebral fractures (∼50% of non-vertebral fracture patients have normal BMD (Schuit et al., 2004)). Cortical bone microstructure (e.g., thickness, porosity) is a more reliable predictor (Samelson et al., 2019), and Wnt16 shows a strong correlation with cortical bone mass. This suggests Wnt16 may serve as an independent predictor of non-vertebral fracture risk, complementing BMD assessments in clinical practice.

### Mechanistic insights: Wnt16 in cortical bone regulation

Wnt16 is predominantly expressed in cortical bone and periosteum, distinguishing it from other Wnt ligands that act on both cortical and trabecular bone. Its dual regulatory effects—promoting osteoblast differentiation (via JNK pathway) and inhibiting osteoclast formation (via OPG-dependent/independent NF-κB pathways)—directly target cortical bone impairment, the key driver of non-vertebral fractures. This mechanism explains why Wnt16 is more effective than traditional therapies for non-vertebral fracture prevention.

### Translational perspectives: drug development

Wnt16 holds promise as a therapeutic target for senile, postmenopausal, and glucocorticoid-induced osteoporosis. Notum inhibitors (e.g., caffeic acid, palmitoleic acid) can enhance Wnt16 signaling by blocking Notum-mediated Wnt degradation (Movérare-Skrtic et al., 2019; Brommage et al., 2019; Zhao et al., 2020), but their bone-specificity requires careful evaluation to avoid systemic over-activation of Wnt signaling in non-skeletal tissues (e.g., liver, kidney). Potential systemic side effects of Wnt16-targeted therapies (e.g., increased cancer risk, metabolic disorders) also warrant further preclinical investigation. Current Wnt16-related drug development is in the preclinical stage, with transgenic mouse models showing promising results (e.g., reduced cortical bone loss in glucocorticoid-treated mice (Ohlsson et al., 2018b)).

## Conclusion

Postmenopausal osteoporosis, as the primary cause of fragility fractures, still has limitations in its current clinical intervention methods, such as having a single target and suboptimal responses in some patients. Therefore, it is urgent to explore new core targets for regulating bone metabolism to optimize the prevention and treatment strategies for non-spinal fractures (Black and Rosen, 2016). The regulation mechanism of osteocyte apoptosis, as an important pathological link in bone mass loss and fracture occurrence in osteoporosis, provides a key direction for targeted intervention and further confirms the clinical value of targeting and regulating bone metabolism pathways in the prevention and treatment of postmenopausal osteoporosis (Epstein, 2007). The core role of the Wnt signaling pathway in regulating bone homeostasis has been clearly demonstrated, and abnormal activation or inhibition of this pathway is closely related to the occurrence of osteoporosis and the increased risk of fractures. This provides an important theoretical basis for the development of anti-osteoporosis drugs targeting this pathway (Grigorie and Leerner, 2018). The mechanical transduction of osteocytes, as a core link in maintaining bone homeostasis, also relies on the key regulation of the Wnt signaling pathway, providing an important mechanism connection for the targeted intervention of mechanical stimulation on bone metabolism and osteoporotic fractures (Almeida et al., 2017). The estrogen receptor signaling pathway, as the core endocrine regulatory pathway for bone metabolism and bone mass maintenance, has built an important mechanism bridge between hormone status and bone health, providing an important reference for analyzing the interaction between the Wnt pathway and the hormone regulatory network (Chamkasem and Toniti, 2015). The meta-analysis of genome-wide association studies has first confirmed a significant correlation between the polymorphism of the WNT16 gene and bone density in premenopausal women, which is a key genetic factor regulating bone strength (Koller et al., 2013). Recent population-based genetic studies have further verified that the polymorphism of the WNT16 gene is closely related to the risk of osteoporotic fractures in postmenopausal women, directly supporting its core role in bone protection (Mitek et al., 2019). Functional knockout experiments have further clarified that the deficiency of the WNT16 gene can significantly reduce cortical bone mass and quality, thereby damaging bone mechanical strength and increasing the susceptibility to fragility fractures. Its specific regulatory effect on cortical bone has become an important entry point for targeted intervention in non-spinal osteoporotic fractures (Hildebrandt et al., 2018), and this regulatory effect is mainly achieved through the classical Wnt signaling pathway in osteoblasts and osteocytes, providing a precise molecular mechanism basis for the specific targeted intervention of WNT16 (Levin et al., 2018). In addition, the long-term anti-fracture efficacy of existing osteoporosis treatment methods still shows significant individual differences, and targeted intervention methods for protecting cortical bone are relatively scarce (Brommage et al., 2019). This review synthesizes evidence that Wnt16 regulates cortical bone density, thickness, and porosity via dual modulation of osteoblasts and osteoclasts, positioning it as a promising therapeutic target for osteoporotic non-vertebral fractures. Key future research priorities include: (1) resolving conflicting findings on Wnt16’s role in glucocorticoid-induced bone loss; (2) validating Wnt16 as a clinical fracture risk predictor; (3) developing bone-specific Notum inhibitors to enhance Wnt16 signaling; (4) evaluating the long-term safety of Wnt16-targeted therapies. These efforts will advance the translation of Wnt16 research into clinical treatments for non-vertebral fractures.
